# New neurons from old beliefs in the adult piriform cortex? A Commentary on: “Occurrence of new neurons in the piriform cortex”

**DOI:** 10.3389/fnana.2015.00062

**Published:** 2015-05-22

**Authors:** Juan Nacher, Luca Bonfanti

**Affiliations:** ^1^Neurobiology Unit and Program in Basic and Applied Neurosciences, Cell Biology Department, Universitat de ValènciaValencia, Spain; ^2^CIBERSAM: Spanish National Network for Research in Mental HealthSpain; ^3^Fundación Investigación Hospital Clínico de Valencia, INCLIVAValencia, Spain; ^4^Neuroscience Institute Cavalieri OttolenghiOrbassano, Italy; ^5^Department of Veterinary Sciences, University of TurinTorino, Italy

**Keywords:** adult neurogenesis, piriform cortex, structural plasticity, PSA-NCAM, doublecortin

In a recent mini-review (Yuan et al., 2015), support is given to the idea that neurons are generated during adulthood in the mammalian piriform cortex (PC), their periventricular origin being also discussed. It is known since long time that a subpopulation of cortical layer II cells in the adult PC of rodents express immature neuronal markers such as polysialylated NCAM (PSA-NCAM; Seki and Arai, 1991; Bonfanti et al., 1992) and doublecortin (DCX; Nacher et al., 2002). These immature neurons have been found in most mammals studied so far, their occurrence being restricted to the paleocortex in rodents (Seki and Arai, 1991; Bonfanti et al., 1992; Nacher et al., 2002), and extended to neocortical areas in species with increasing brain size and gyrencephaly, e.g., rabbits, cats, and primates (Bonfanti and Nacher, 2012). The fact that these cortical cells express markers of immaturity which are usually present in the young neurons produced within adult neurogenic sites (Bonfanti and Theodosis, 1994; Brown et al., 2003), suggested the possibility that they might be newly generated. Across the years, different laboratories undertook 5-bromo-2-deoxyuridine (BrdU) pulse-chase experiments to test this hypothesis. A first group of studies reported that some of the cells in the PC layer II have been generated during adulthood (Bernier et al., 2002; Pekcec et al., 2006; Shapiro et al., 2007a,b). However, the number of recently generated neurons was extremely low, they appeared to have a transient existence, and their precise location in the PC was not properly indicated. In parallel, many other studies did not find evidence for the incorporation of new neurons in the PC of adult rats (Nacher et al., 2002; Gomez-Climent et al., 2008), rabbits (Luzzati et al., 2009), and cats (Varea et al., 2011). These latter studies by no means exclude the possibility of adult neurogenesis in this area; yet, for their relevance, they should be reported in any review article dealing with such controversial issue. By contrast, in their mini-review Yuan et al. deliberately chose to ignore these articles, together with those that have provided solid evidence for the embryonic origin of most of these cells (Gomez-Climent et al., 2008), suggesting that they represent a “reservoir” of non-newly generated, immature neurons which maintain features of structural plasticity throughout life (Bonfanti and Nacher, 2012).

In addition, they omit several important data. First of all, the well-established fact that PSA-NCAM and DCX can also be expressed by non-newly generated cells, which perform other forms of structural plasticity (Bonfanti, 2006; Bonfanti and Nacher, 2012). When discussing the fate of the PC immature neurons they omit that these cells progressively disappear as aging progresses (Abrous et al., 1997; Murphy et al., 2001; Xiong et al., 2008; Varea et al., 2009). Additionally, they cite the work by Nacher et al. (2004) as describing reduction in PC neurogenesis after chronic stress, when only PSA-NCAM expression was studied herein. Again, the expression of PSA-NCAM by cells of the adult brain is mistaken for the occurrence of adult neurogenesis. Finally, they make statements which are not supported by the current literature, e.g., by affirming that many of the immature neurons can be classified as neurogliaform cells. The small cells expressing immature neuronal markers in the PC were erroneously classified as neurogliaform cells, an interneuronal subtype (Price et al., 2005), but later phenotypic analysis clearly demonstrated that they were not mature interneurons (Gomez-Climent et al., 2008).

Concerning the putative fate of these cells, the Authors only cite work by Bedard and Parent (2004) and Klempin et al. (2011), indicating that these studies found some DCX+ cells coexpressing interneuronal markers. They claim that both studies used DCX-GFP mice lineage tracing, which only was used by Klempin et al. (2011). This study has to be taken cautiously because, as acknowledged by the authors, a fraction of GFP-expressing cells did not express DCX protein. The study of Bedard and Parent is focused on olfactory bulb neurogenesis in humans, and does not contain any reference to adult neurogenesis in the PC. Here again the Authors decide to ignore the more solid evidence regarding the putative nature and fate of the PC immature neurons, which revealed that many of them have the typical structure of PC principal cells (Gomez-Climent et al., 2008) and express molecules exclusively found in pallial-derived excitatory neurons, another aspect playing against an origin from the SVZ (Gomez-Climent et al., 2008; Luzzati et al., 2009). Yuan et al. also mention the possibility that the recently generated neurons differentiate into pyramidal neurons, citing the work by Guo et al. (2010). However, this study (as also reported in Rivers et al., 2008) proposed that these neurons could be generated by NG2+ local glial progenitors which have ceased to proliferate at least since the first postnatal month, then undergoing maturation (Rivers et al., 2008; Guo et al., 2010); but technical artifacts might have led to misinterpretation of the results, as acknowledged by the same Authors (Richardson et al., 2011). A complex set of experiments carried out in these studies clearly excluded that new neurons can reach the PC through migration from the SVZ neurogenic site. In spite of these data, completely omitted by Yuan et al., a schematic figure of their mini-review represents the putative newlyborn PC neurons as coming from the SVZ through a migratory stream. The image is described as modified from Klempin et al. (2011), yet, it is not the graphics but the significance of the figure itself which has been modified, since a non-neurogenic PC in the original article by Klempin et al., has been now turned into a neurogenic region (Figure 1).

**Figure 1:**
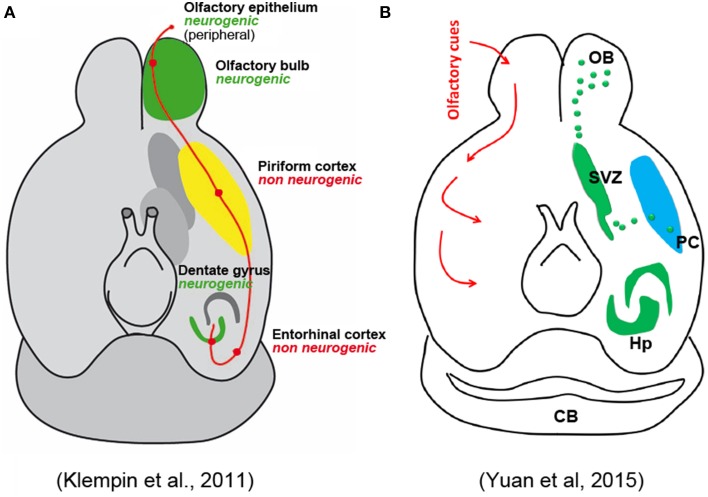
**Schematic representation of olfactory pathways and relative neurogenic and non-neurogenic areas in the adult brain of rodents. (A)** Original figure by Klempin et al. (2011); **(B)** figure in Yuan et al. (2015) reported as “modified” from the Klempin paper. Note that the non-neurogenic piriform cortex in **(A)** has became neurogenic in **(B)** with newlyborn cells coming from the SVZ through a caudal migratory stream.

On the whole, the article by Yuan et al. fails to acknowledge that some of the published evidence for this phenomenon is disputable, that many papers in the literature denied it, and that, if existent, it appears to be a very restricted/unusual event (Bonfanti and Peretto, 2011), thus leaving the reader with the false impression that most of the PC immature neurons might be generated postnatally.

## Conflict of interest statement

The authors declare that the research was conducted in the absence of any commercial or financial relationships that could be construed as a potential conflict of interest.
